# Urea deep placement reduces yield-scaled greenhouse gas (CH_4_ and N_2_O) and NO emissions from a ground cover rice production system

**DOI:** 10.1038/s41598-017-11772-2

**Published:** 2017-09-12

**Authors:** Zhisheng Yao, Xunhua Zheng, Yanan Zhang, Chunyan Liu, Rui Wang, Shan Lin, Qiang Zuo, Klaus Butterbach-Bahl

**Affiliations:** 1 State Key Laboratory of Atmospheric Boundary Layer Physics and Atmospheric Chemistry, Institute of Atmospheric Physics, Chinese Academy of Sciences, Beijing, 100029 P.R. China; 2 Institute for Meteorology and Climate Research, Atmospheric Environmental Research, Karlsruhe Institute of Technology, D-82467 Garmisch-Partenkirchen, Germany; 3 College of Earth Science, University of Chinese Academy of Sciences, Beijing, 100049 P.R. China; 4 College of Resource and Environmental Science, China Agricultural University, Beijing, 100193 P.R. China

## Abstract

Ground cover rice production system (GCRPS), i.e., paddy soils being covered by thin plastic films with soil moisture being maintained nearly saturated status, is a promising technology as increased yields are achieved with less irrigation water. However, increased soil aeration and temperature under GCRPS may cause pollution swapping in greenhouse gas (GHG) from CH_4_ to N_2_O emissions. A 2-year experiment was performed, taking traditional rice cultivation as a reference, to assess the impacts of N-fertilizer placement methods on CH_4_, N_2_O and NO emissions and rice yields under GCRPS. Averaging across all rice seasons and N-fertilizer treatments, the GHG emissions for GCRPS were 1973 kg CO_2_-eq ha^−1^ (or 256 kg CO_2_-eq Mg^−1^), which is significantly lower than that of traditional cultivation (4186 kg CO_2_-eq ha^−1^or 646 kg CO_2_-eq Mg^−1^). Furthermore, if urea was placed at a 10–15 cm soil depth instead of broadcasting, the yield-scaled GHG emissions from GCRPS were further reduced from 377 to 222 kg CO_2_-eq Mg^−1^, as N_2_O emissions greatly decreased while yields increased. Urea deep placement also reduced yield-scaled NO emissions by 54%. Therefore, GCRPS with urea deep placement is a climate- and environment-smart management, which allows for maximal rice yields at minimal GHG and NO emissions.

## Introduction

Trace gas emissions from agricultural soils, specifically methane (CH_4_), nitrous oxide (N_2_O) and nitric oxide (NO), are of key relevance for atmospheric photochemistry and global climate change^1^. Upland crop systems primarily release N_2_O and NO, while waterlogged paddy rice systems are significant sources for atmospheric CH_4_ and the N-trace gases (N_2_O and NO)^2, 3^. Greenhouse gas (GHG) emissions from paddy rice production systems are estimated to contribute approximately 55% of the worldwide budget of GHG emissions from agricultural soils^1^. In a recent meta-analysis study, Linquist *et al*.^4^ provided evidence that GHG (CH_4_ and N_2_O) emissions from paddy rice cultivations are roughly four times higher than those originating from the production of other cereals such as wheat or maize. In view of such high GHG emissions from rice production systems, there is strong interest in its mitigation. However, as most CH_4_ mitigation strategies for rice production systems are based on water management and periodical soil aeration, trade-offs with regard to N-trace gas (namely N_2_O and NO) emissions, which might be stimulated under increased soil aeration, need to be considered to avoid significant pollution swapping.

China presently cultivates approximately 30 million ha of rice per year and is the world’s largest rice producer^5^. In recent decades, however, rice production in China is facing unprecedented challenges, including the increasing demand of its growing population, losses of cropping area to infrastructure development, massive increases in water consumption by booming industries and on-going climate change that affects irrigation water supply^6^. Thus, rice production systems need to be more productive while at the same time its environmental footprint, i.e., irrigation water demand and soil GHG emissions, needs to be reduced. One of the most promising technologies and management practices to overcome water shortages and increase rice production, already put in practice on more than 4 million ha within China, is the so-called ground cover rice production system (GCRPS), particularly with transplanted rice seedlings^7–9^. Here strip soil beds are covered with thin plastic films and soil water content is kept close to saturation via irrigation-furrows between raised beds, thus avoiding standing water. The GCRPS-induced alteration from flooded anaerobic paddy soils to more aerated soil conditions may result in pollution swapping, i.e., reductions of CH_4_ emissions at the expense of increases in N_2_O emissions^10^. For example, Kreye *et al*.^11^ reported that compared to flooded rice cultivations, GCRPS with direct seeding showed comparable or higher total GHG (CH_4_ and N_2_O) emissions due to considerably increased N_2_O emissions. In contrast, Yao *et al*.^6^ observed that although GCRPS with transplanted rice seedlings actually increased N_2_O emissions, total GHG emissions were decreased. As CH_4_ emissions in GCRPS systems are already low, total GHG mitigation must be achieved by a reduction in N_2_O emissions, which points towards improved N fertilizer management.

Generally, incorporating N fertilizer more deeply into the soil and/or placing it in concentrated bands instead of uniformly broadcasting it to the soil surface as presently done in GCRPS^8^, are considered as good agricultural practices, as they may reduce NH_3_ and aerobic microbial process (e.g., nitrification of NH_4_^+^ to NO_3_^−^), improve root zone access to nutrient N and increase overall crop N use efficiency^12^. Also, deep placement of N fertilizers has been observed to play a vital role in regulating CH_4_, N_2_O and NO fluxes. For example, field and laboratory studies report variable (inhibitory, stimulatory or no difference) results on the effect of N deep placement on CH_4_ emission^13–15^ and emissions of N_2_O and NO, particularly N_2_O from upland crop systems^12, 16–19^. The various results may be caused by different interactions of soil N availability and site-specific conditions. Recently, deep placement of N fertilizer (mainly urea) is gaining popularity for rice cultivation in the Asian countries as significant increases in yields can be achieved^20^. However, little is known about how this practice is affecting GHG and NO emissions^15, 20^ and nothing is known for GCRPS.

In this study, we present the results of a 2-year field measurement in which CH_4_, N_2_O and NO fluxes as well as rice yields were determined simultaneously for the water-saving ground cover rice production system as well as for the traditional rice cultivation to identify potential GHG mitigation effects of urea deep placement. We hypothesized that GCRPS under urea broadcast treatment is better in terms of yields and yield-scaled GHG emissions as compared to the corresponding traditional rice cultivation and that urea deep placement for GCRPS can even further lower GHG emissions while increasing yields. As we hypothesized that plant N use efficiency is increasing with urea deep placement, we also postulated that yield-scaled NO emissions will be lower for this management practice.

## Results

### Environmental variables and rice yields

**Figure 1:**
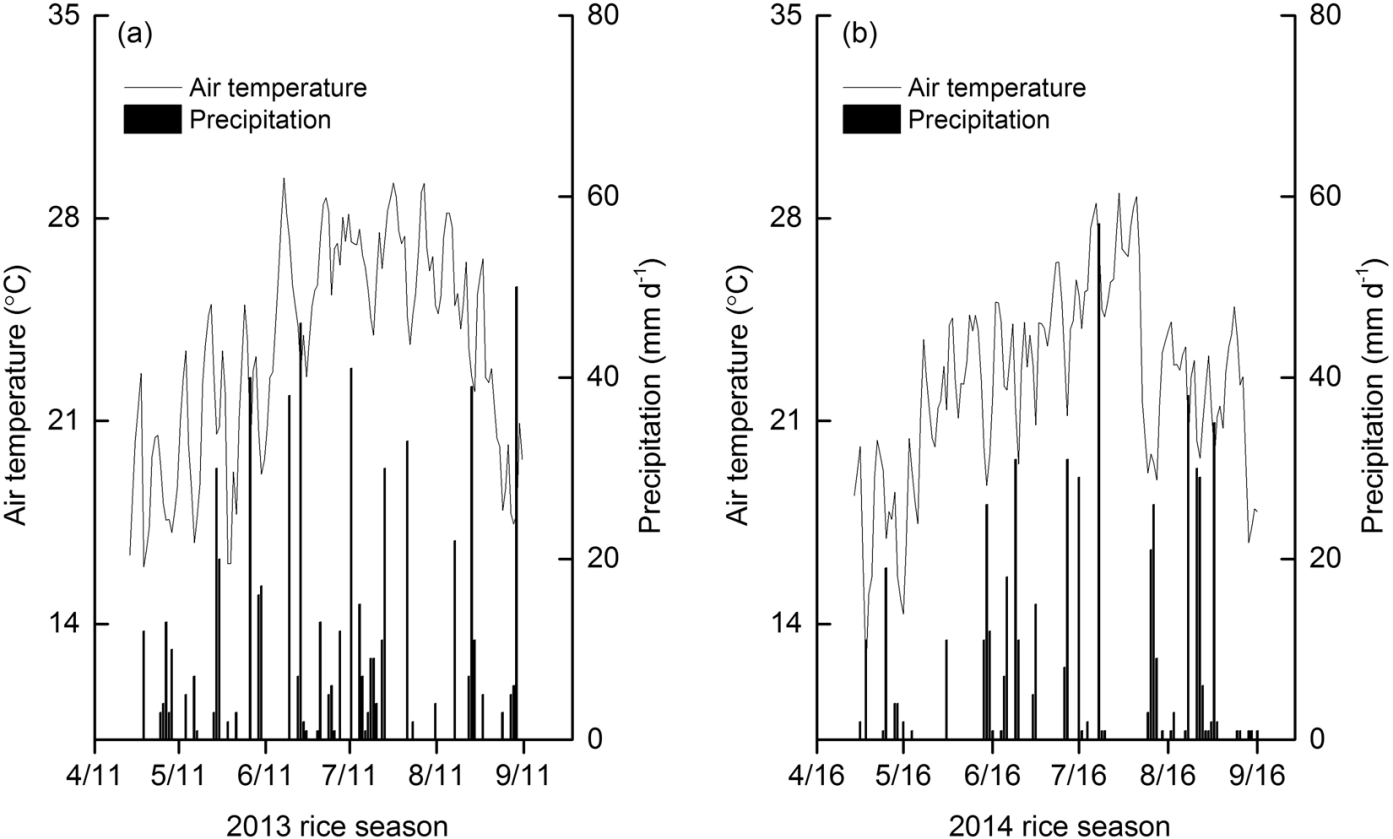
Seasonal variations of mean daily air temperature and daily cumulative precipitation during the rice-growing seasons of 2013 and 2014.

**Figure 2:**
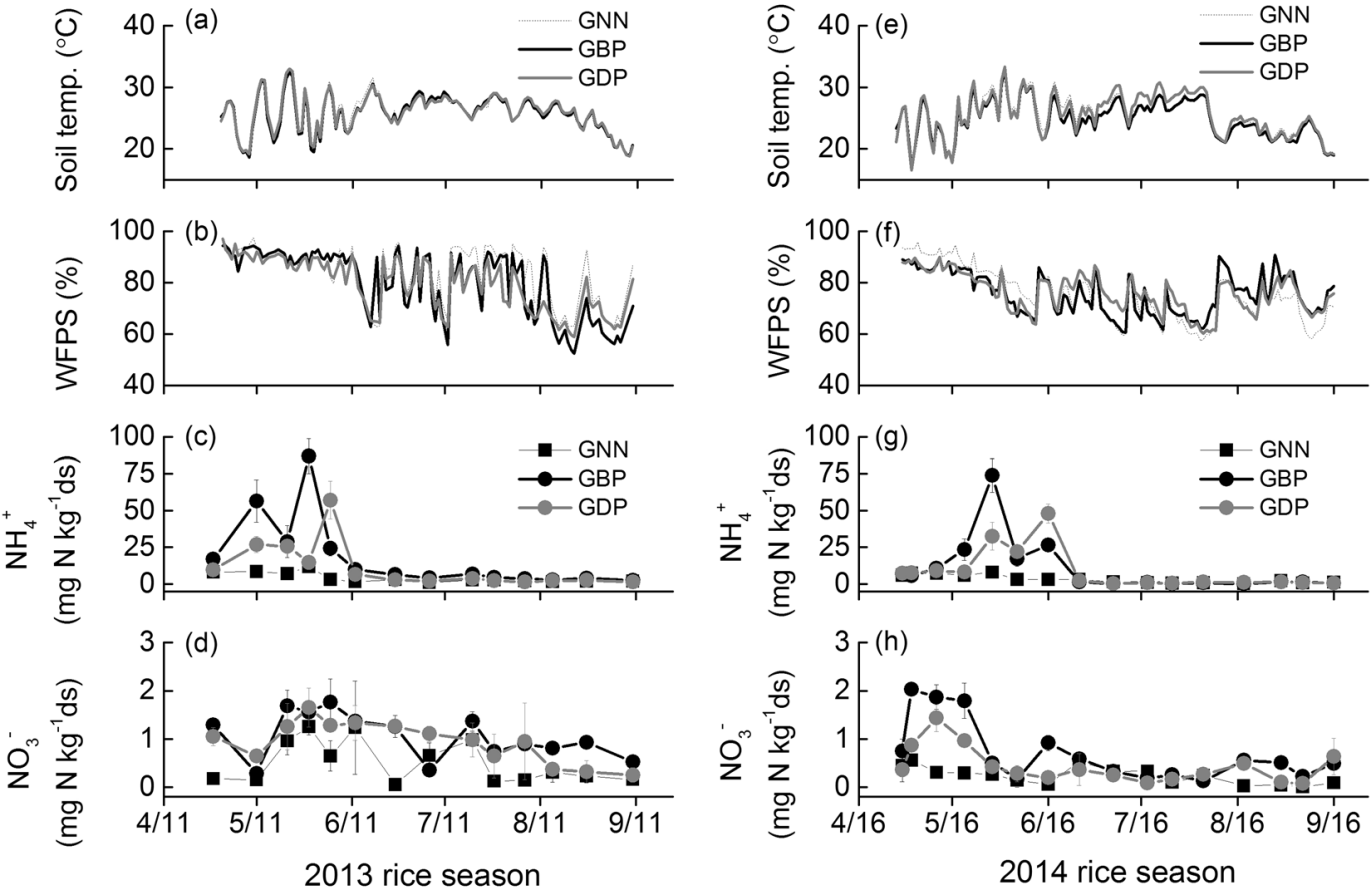
Seasonal variations of (**a**,**e**) soil (5 cm) temperature, (**b**,**f**) soil (0–6 cm) water content expressed as water-filled pore space (WFPS), and soil (**c**,**g**) ammonium (NH_4_^+^) and nitrate (NO_3_^−^) concentrations in the ground cover rice production system under different fertilizer treatments (i.e., no N fertilization (GNN), broadcast placement of urea (GBP) and deep-point placement of urea (GDP)) during the rice-growing seasons of 2013 and 2014.

Neither air temperature nor soil temperature differed significantly between two rice-growing seasons or urea placement methods (Figs 1 and 2a,e). Air temperature ranged from 12.4 to 29.4 °C, while the range of mean soil temperatures was 24.7–26.0 °C. In contrast, total seasonal rainfall was 638 mm in 2013, which was approximately 19% higher than in 2014. In both seasons, the urea placement methods did not affect soil water content expressed as WFPS (water-filled pore space) (Fig. 2b,f). The mean WFPS across the rice-growing season was 79.7–84.2% and 75.3–75.9% for 2013 and 2014, respectively, with the highest in GNN (i.e., no N fertilization of the ground cover rice production system) for both seasons.

During the rice-growing season, soil NH_4_^+^, the dominant form of mineral N in GCRPS systems with broadcast (GBP) or deep placement of urea (GDP) at a common rate of 150 kg N ha^−1^ showed substantial peaks following urea applications (Fig. 2c,g). Nevertheless, the NH_4_^+^ concentrations in GBP were generally higher than those of GDP for approximately one month after fertilization. Also, the maximum NH_4_^+^ concentrations in GBP appeared earlier as compared to GDP (May 28 vs. June 4, 2013; May 29 vs. June 16, 2014). Soil NO_3_^−^ concentrations in all treatments were generally below 2 mg N kg^−1^ds (dry soil) throughout the experiment, but they also showed the tendency of GBP > GDP > GNN (Fig. 2d,h).

**Figure 3:**
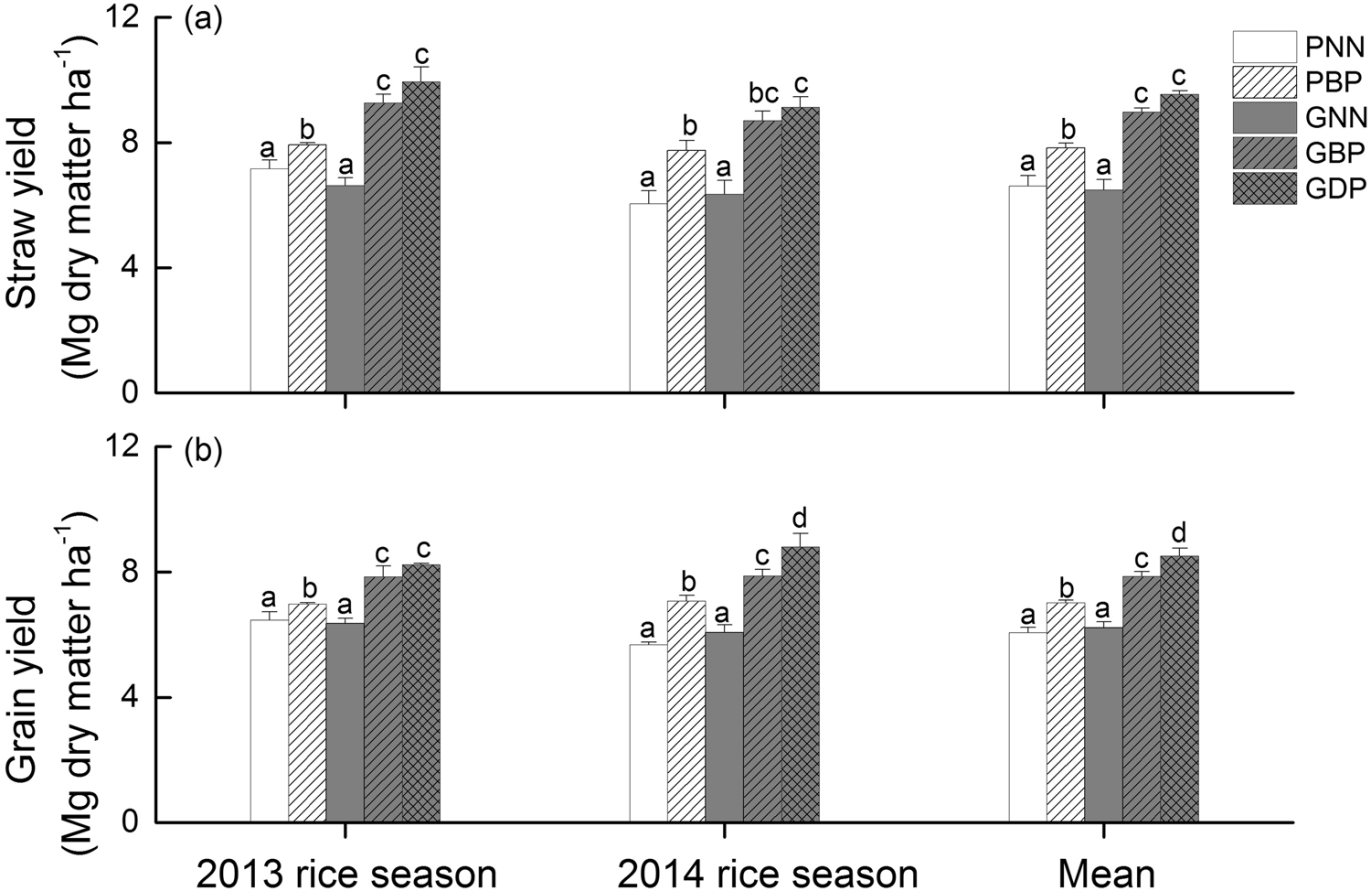
Yields of (**a**) straw and (**b**) grain at physiological maturity in the different treatments of combined rice cultivation and N fertilizer for 2013 and 2014 rice seasons. “Mean” stands for mean values of two rice-growing seasons of 2013 and 2014. PNN, no N fertilization in the traditional paddy rice production system; PBP, broadcast placement of urea in the traditional paddy rice production system; GNN, no N fertilization in the ground cover rice production system; GBP, broadcast placement of urea in the ground cover rice production system; GDP, deep-point placement of urea in the ground cover rice production system. For each season, bars with different letters indicate significant differences among N fertilizer treatments at the P < 0.05 level.

Broadcasting urea in the traditional rice cultivation (PBP) resulted in straw and grain yields of 7.74–7.93 and 6.98–7.07 Mg dry matter ha^−1^, respectively. Compared to PBP, on average, GBP significantly increased straw and grain yields by 15% and 12%, respectively. Straw and grain yields for GDP were even 22% and 21% higher as compared to PBP, respectively (Fig. 3).

### CH_4_ emissions

**Figure 4:**
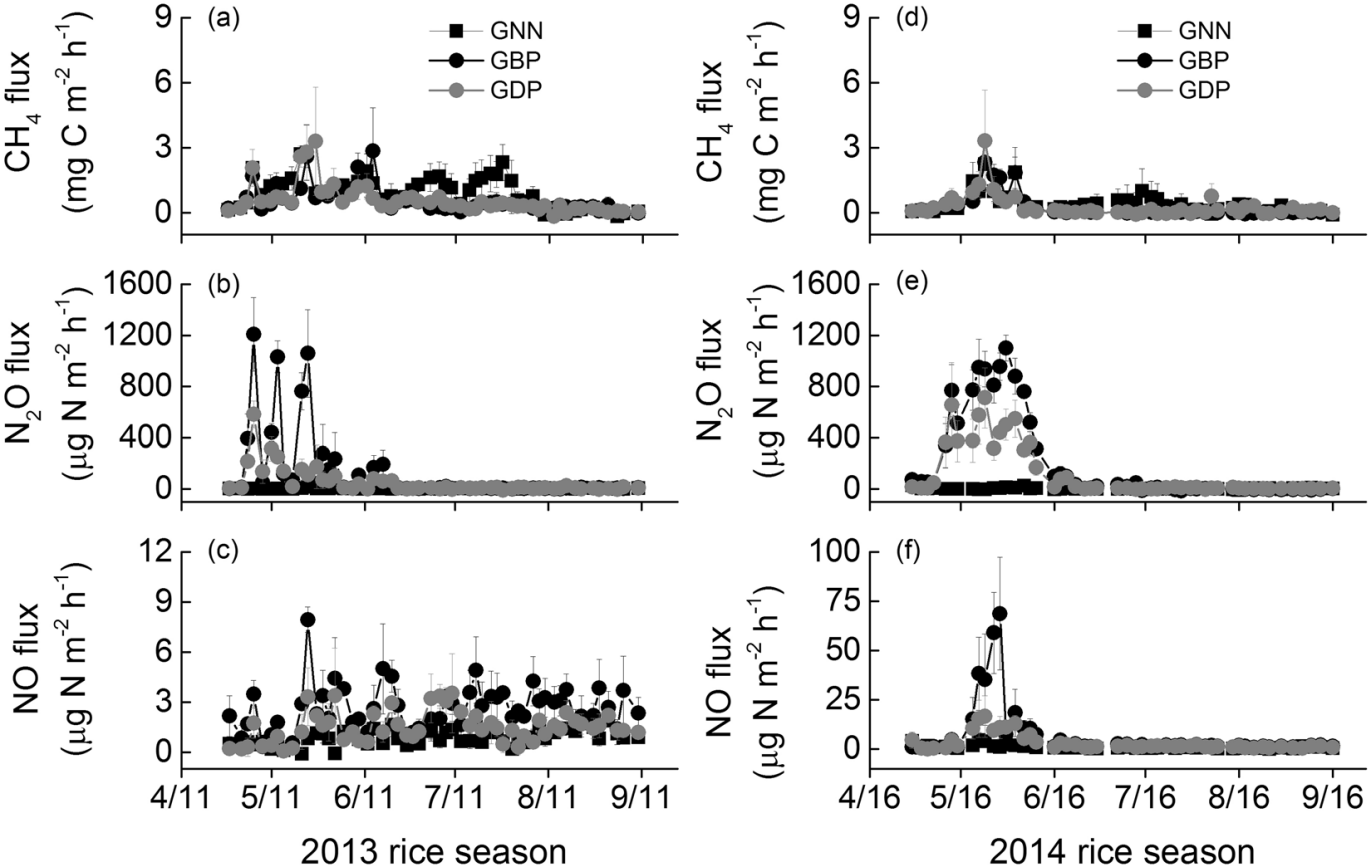
Seasonal variations of (**a**,**d**) methane (CH_4_), (**b**,**e**) nitrous oxide (N_2_O), and (**c**,**f**) nitric oxide (NO) fluxes from the ground cover rice production system under different fertilizer treatments (i.e., no N fertilization (GNN), broadcast placement of urea (GBP) and deep-point placement of urea (GDP)) during the rice-growing seasons of 2013 and 2014.

CH_4_ emissions from the traditional rice cultivation (i.e., PNN and PBP) continuously increased following field flooding and peaked in mid-August, 2013 (10.3–18.5 mg C m^−2^ h^−1^) or in mid-July, 2014 (8.5–9.3 mg C m^−2^ h^−1^). Thereafter, CH_4_ emissions tended to decline with emissions approaching zero towards the final drain (see the Supplementary Information Fig. S1). As expected, CH_4_ emissions in GCRPS were low with only a few minor emission pulses (<3.5 mg C m^−2^ h^−1^) during the high WFPS period. Fluxes were negligible during periods of field drying and following final drainage for harvest (Fig. 4a,d).

Over the rice-growing season, cumulative CH_4_ emissions were significantly affected by the rice production system, but were independent of the N fertilizer treatment, year and the interaction of these factors (see the Supplementary Information Table S1). As a result, total seasonal CH_4_ emissions under GCRPS were on average 80% lower as compared to the traditional rice cultivation. Among all GCRPS plots, the urea placement methods showed no distinct influences on either area- or yield-scaled CH_4_ emissions (Tables 1 and 2). During the fallow period of 2013–2014, all treatments were a weak sink of atmospheric CH_4_ (0.55–1.02 kg C ha^−1^, see the Supplementary Information Table S2).

**Table 1:** Seasonal cumulative emissions of methane (CH_4_, in kg C ha^−1^), nitrous oxide (N_2_O, in kg N ha^−1^) and nitric oxide (NO, in kg N ha^−1^) and direct emission factors of N_2_O (EF-N_2_O, in %) and NO (EF-NO, in %) for various agronomic treatments during the rice-growing seasons of 2013 and 2014.

Season	Code^†^	CH_4_*	N_2_O*	EF-N_2_O	NO*	EF-NO
2013 rice season						
	PNN	71.3 ± 13.4a	0.19 ± 0.05a		0.022 ± 0.002a	
	PBP	82.7 ± 7.1a	1.97 ± 0.16b	1.18	0.053 ± 0.009b	0.021
	GNN	30.4 ± 9.1b	0.18 ± 0.03a		0.028 ± 0.004a	
	GBP	18.3 ± 5.8b	3.77 ± 0.50c	2.39	0.086 ± 0.017b	0.039
	GDP	19.0 ± 4.3b	1.63 ± 0.25b	0.97	0.049 ± 0.001b	0.014
2014 rice season						
	PNN	96.0 ± 31.1a	0.10 ± 0.03a		0.015 ± 0.002a	
	PBP	77.0 ± 8.8a	1.85 ± 0.44b	1.17	0.027 ± 0.001a	0.008
	GNN	13.3 ± 7.8b	0.11 ± 0.03a		0.032 ± 0.003a	
	GBP	7.6 ± 1.2b	6.41 ± 0.64c	4.20	0.200 ± 0.099b	0.112
	GDP	8.4 ± 3.1b	3.79 ± 0.67b	2.46	0.098 ± 0.027b	0.044
Mean of 2013 and 2014						
	PNN	83.6 ± 22.2a	0.14 ± 0.04a		0.018 ± 0.001a	
	PBP	79.9 ± 3.5a	1.91 ± 0.29b	1.18	0.040 ± 0.005b	0.014
	GNN	21.8 ± 1.0b	0.14 ± 0.03a		0.030 ± 0.002a	
	GBP	13.0 ± 2.5b	5.09 ± 0.44c	3.30	0.143 ± 0.041c	0.075
	GDP	13.7 ± 1.8b	2.71 ± 0.22b	1.71	0.073 ± 0.013bc	0.029

^†^PNN, no N fertilization in the traditional paddy rice production system; PBP, broadcast placement of urea at a common rate of 150 kg N ha^−1^ in the traditional paddy rice production system; GNN, no N fertilization in the ground cover rice production system; GBP, broadcast placement of urea at a common rate of 150 kg N ha^−1^ in the ground cover rice production system; GDP, deep-point placement of urea at a common rate of 150 kg N ha^−1^ in the ground cover rice production system. *Different letters within the same column indicate significant differences among N fertilizer treatments in each rice season at the P < 0.05 level.

**Table 2:** Grain yield-scaled seasonal emissions of methane (CH_4_, in kg C Mg^−1^), nitrous oxide (N_2_O, in g N Mg^−1^) and nitric oxide (NO, in g N Mg^−1^) for various agronomic treatments during the rice-growing seasons of 2013 and 2014.

Code^†^	Yield-scaled emissions (2013 rice season)*			Yield-scaled emissions (2014 rice season)*			Yield-scaled emissions (mean of 2013 and 2014)*		
	CH_4_	N_2_O	NO	CH_4_	N_2_O	NO	CH_4_	N_2_O	NO
PNN	11.2 ± 2.5a	29.3 ± 8.4a	3.4 ± 0.2a	16.9 ± 5.4a	17.3 ± 4.7a	2.6 ± 0.3a	14.0 ± 3.9a	23.3 ± 6.4a	3.0 ± 0.1a
PBP	11.8 ± 0.9a	282 ± 21b	7.6 ± 1.2bc	10.9 ± 1.4a	263 ± 65b	3.8 ± 0.2a	11.4 ± 0.6a	272 ± 40b	5.7 ± 0.7bd
GNN	4.7 ± 1.3b	27.8 ± 3.9a	4.4 ± 0.6ac	2.3 ± 1.4b	17.4 ± 4.1a	5.2 ± 0.5b	3.5 ± 0.1b	22.6 ± 4.0a	4.8 ± 0.4ab
GBP	2.3 ± 0.6b	486 ± 78c	11.2 ± 2.5b	1.0 ± 0.1b	812 ± 72c	25.8 ± 13.3c	1.6 ± 0.3b	649 ± 48c	18.5 ± 5.4c
GDP	2.3 ± 0.5b	198 ± 30b	5.9 ± 0.2bc	1.0 ± 0.1b	432 ± 77b	11.0 ± 2.7bc	1.6 ± 0.2b	315 ± 28b	8.5 ± 1.3d

^†^Codes of the combined rice cultivation and N fertilizer treatments are defined in the footnotes of Table 1 and in the text. *Different letters within the same column indicate significant differences among N fertilizer treatments in each rice season at the P < 0.05 level.

### N_2_O emissions

Significant N_2_O emissions from rice paddies were observed mainly during the first month (traditional rice cultivation) or within the first 6 weeks (GCRPS) after urea applications, while thereafter emissions were mostly low (Fig. 4b,e and S1). The cumulative N_2_O emissions across the rice-growing season accounted for 72–84% of total annual emissions in GBP and GDP treatments (Table 1 and S2). But total N_2_O emissions during the rice-growing season varied significantly with the rice production system, N fertilizer treatment, year and their interactions (see the Supplementary Information Table S1). When urea was broadcast, seasonal N_2_O emission under GCRPS (GBP) was 3.77–6.41 kg N ha^−1^, which was significantly higher compared to the traditional rice cultivation (PBP: 1.91–1.97 kg N ha^−1^). However, the deep placement of urea (GDP) significantly reduced N_2_O emissions by 47% on average as compared to GBP. There were no significant differences between GDP and PBP (Table 1). Across the rice seasons, the trend and magnitude of urea placement methods effects on the yield-scaled N_2_O emissions were comparable to their impacts on the area-scaled N_2_O (Table 2). During the fallow period of 2013–2014, total N_2_O emissions ranged from 0.48 to 2.29 kg N ha^−1^, with no significant differences between treatments (see the Supplementary Information Table S2).

Over the two rice seasons, direct emission factors of N_2_O for GBP were on average 181% higher as compared to PBP. However, urea deep placement in GCRPS (GDP) decreased the N_2_O emission factor to the level of that observed for PBP (Table 1).

### NO emissions

NO emission pulses occurred following urea application or during the dry period (Fig. 4c,f and S1). Overall, NO emissions were mostly very low (<5 µg N m^−2^ h^−1^) in all treatments. Only for GBP a substantial emission pulse (68.7 µg N m^−2^ h^−1^) was observed at the end of May, 2014. In contrast to N_2_O emission, seasonal NO emissions from all treatments were generally low, ranging from 0.015 to 0.200 kg N ha^−1^ (Table 1). Statistical analysis indicated that total seasonal NO emissions were significantly affected by the rice production system and N fertilizer treatment, whereas the year and the interaction of these factors had no significant effects (see the Supplementary Information Table S1). For the two rice seasons, NO emissions from GBP were consistently higher than those of PBP (2013: 62%; 2014: 651%) (Table 1). Nevertheless, urea deep placement (GDP) reduced the area- and yield-scaled NO emissions by 49% and 54%, respectively, as compared to GBP (Tables 1 and 2). For the fallow season of 2013–2014, there was no significant difference in NO emission between treatments (range: 0.11–0.17 kg N ha^−1^) (see the Supplementary Information Table S2).

During the rice-growing seasons, stepwise multiple linear regression analysis showed a significant relationship between N_2_O + NO fluxes and soil inorganic N (NH_4_^+^ + NO_3_^−^) concentrations for both GBP and GDP treatments (i.e., GBP in the 2013 season: ${F}_{{N}_{2}O+NO}=\,5.57\times (N{H}_{4}^{+}+N{O}_{3}^{-})+19.9,\,{R}^{2}=0.36,\,P < 0.05$; GBP in the 2014 season: ${F}_{{N}_{2}O+NO}=\,16.2\,\times (N{H}_{4}^{+}+N{O}_{3}^{-})+41.6,\,{R}^{2}=0.77,\,P < 0.05$; GDP in the 2013 season: ${F}_{{N}_{2}O+NO}=\,3.51\times (N{H}_{4}^{+}+N{O}_{3}^{-})+\,5.00,\,{R}^{2}=0.39,\,P < 0.05$ and GDP in the 2014 season: ${F}_{{N}_{2}O+NO}=\,10.1\,\times (N{H}_{4}^{+}+N{O}_{3}^{-})+\,27.1,\,{R}^{2}=0.61,\,P < 0.05$).

Averaged over two rice seasons, direct emission factors of NO were 0.075%, 0.029% and 0.014% for GBP, GDP and PBP, respectively (Table 1).

### Total GHG (CH_4_ + N_2_O) emissions

**Figure 5:**
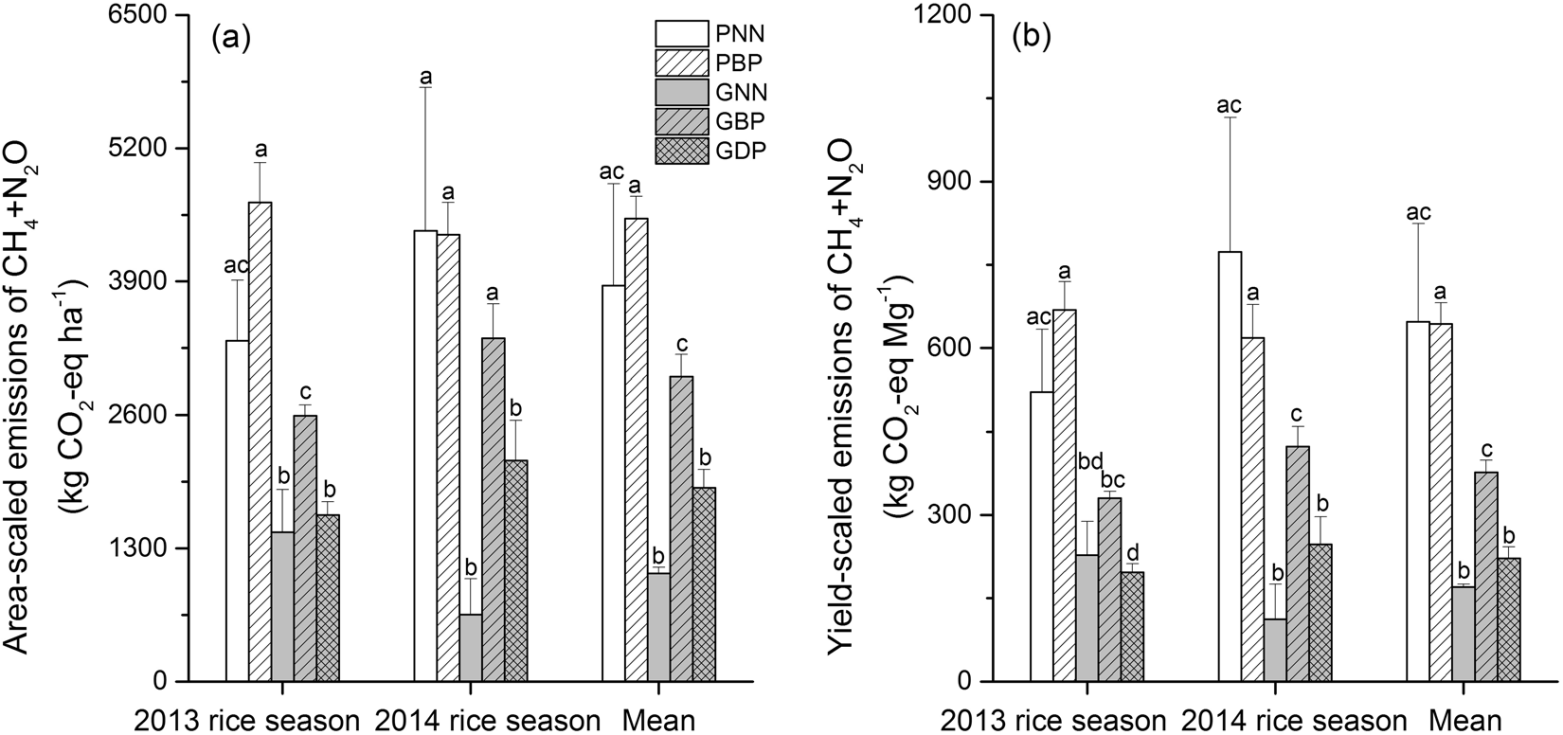
Seasonal area- and yield-scaled carbon dioxide (CO_2_) equivalents of methane (CH_4_) plus nitrous oxide (N_2_O) emissions for the different rice cultivation and N fertilizer treatments during the 2013 and 2014 rice seasons. “Mean” represents mean values of the two rice-growing seasons of 2013 and 2014. PNN, no N fertilization of the traditional paddy rice production system; PBP, broadcast placement of urea in the traditional paddy rice production system; GNN, no N fertilization in the ground cover rice production system; GBP, broadcast placement of urea in the ground cover rice production system; GDP, deep-point placement of urea in the ground cover rice production system. For each season, bars with different letters indicate significant differences among N fertilizer treatments at the P < 0.05 level.

Seasonal aggregated emissions of CH_4_ and N_2_O for all treatments ranged from 654 to 4672 kg CO_2_-eq ha^−1^ when expressed on an area basis and from 112 to 774 kg CO_2_-eq Mg^−1^ when expressed on a grain yield basis (Fig. 5). Seasonal CH_4_ + N_2_O emissions depended greatly on the rice production system and N fertilizer treatment, but the totals were not significantly affected by the year and the interaction of these factors (see the Supplementary Information Table S1). Compared to the traditional rice cultivation (PBP), GCRPS with urea broadcast (GBP) or deep placement (GDP) significantly reduced total GHG emissions, irrespective of their expression on either the area- or yield-scaled basis (Fig. 5). Furthermore, the area- and yield-scaled total GHG emissions could be reduced by 36% and 41%, respectively, by changing urea broadcast to deep placement in GCRPS (GBP vs. GDP).

## Discussion

It is well accepted that management of N fertilizer and irrigation water can influence production of biogenic gases (CH_4_, N_2_O and NO). Compared to the traditional rice cultivation, both GBP and GDP significantly reduced CH_4_ emissions, but there was no significant difference in either area- or yield-scaled CH_4_ emissions between GBP and GDP (Tables 1 and 2). Similarly, Adviento-Borbe & Linquist^15^ observed that there was no influence of urea placement methods on daily CH_4_ fluxes and cumulative emissions in three rice field trials. In contrast, Linquist *et al*.^14^ reported reduction in CH_4_ emissions with urea deep placement in comparison to urea broadcast application in rice fields. They proposed that both concentrating NH_4_^+^ into localized areas and increasing oxygen availability in the rhizosphere would likely stimulate CH_4_ oxidation by soil methanotrophs and reduce overall emissions^14^. In this study, more prevailing oxidized soil conditions in GCRPS (as indicated by the range of observed WFPS: 75–84%) obviously reduced CH_4_ production by inhibiting methanogenesis and/or increased CH_4_ consumption by stimulating methane-oxidizing bacteria^6^. Thus, the effect of soil water regime was the major driver of low CH_4_ emissions, thereby overriding possible effects of urea placement methods on CH_4_ emissions.

On the other hand, averaging across two rice seasons, GCRPS significantly increased N_2_O and NO emissions by 167% and 259% relative to traditional rice cultivation, respectively, when urea was broadcast (GBP vs. PBP). However, the deep placement of urea in GCRPS (GDP) significantly decreased N_2_O and NO emissions as compared to GBP, and showed comparable levels to PBP (Table 1). A number of studies on upland crop systems reported that N deep placement generally reduced NO emissions^16, 17, 21^, which is in agreement with our present findings; but this practice tended to increase N_2_O emissions from upland crop systems, as a result of significant soil NO_2_^−^ accumulations inducing generation of substantial N_2_O through biologic nitrifier-denitrification and abiotic processes (e.g., chemo-denitrification)^17, 18, 21, 22^. In addition, a few studies on rice fields observed that the effect of N deep placement on N_2_O emissions could be either inhibitory^20^, stimulatory^23^ or did not show any significant difference^15^. Only in one study the effect of urea deep placement on NO emissions from rice systems was investigated and the study concluded that NO emissions were negligible and not affected by N placement methods^20^. Also in our study NO emissions were relatively low as total seasonal emissions were <0.3 kg N ha^−1^, which is at the lowest end of emissions from cultivated temperate and tropical soils with average emissions ranging from 0.44 to 2 kg N ha^−1^ yr^−1^ ^24, 25^. Given the diversity of effects of N fertilizer placement and type on soil processes and plant uptake and in view of the huge variability of agricultural systems, it was postulated that soil N availability interacts with other site-specific factors (e.g., soil properties, climate conditions or agricultural practices) to regulate processes of N_2_O and NO production, consumption and release. This complexity might result in contradictory results about the effects of N deep placement on N-trace gas emissions. As observed in our study, soil NH_4_^+^ and NO_3_^−^ concentrations across two rice seasons were usually lower in GDP than in GBP (Fig. 2), which was likely tied to relatively slow release of NH_4_^+^ (see the temporal dynamics of soil NH_4_^+^ in Fig. 2) and/or higher crop N uptake in GDP (as indicated by crop productivity). Thus, the inhibitory effect of GDP on N_2_O and NO emissions was probably ascribed to the fact that GDP decreased the supply of mineral N substrates within 0–10 cm soil layer as the placement was targeted at 10–15 cm soil depth. However, the 0–10 cm soil horizon is commonly seen as the most biologically active zone, also with regard to microbial production processes of N_2_O and NO^26^. Moreover, in 10–15 cm soil depth, the soil water content is higher relative to the topsoil, and thus more anaerobic. This not only hampers the oxidation of urea or ammonium to nitrate by nitrification, but also increases N_2_O and NO diffusion transport through soil to atmosphere and promotes the complete reduction of oxidized mineral N compounds to N_2_ via denitrification^27, 28^.

In our study, the fertilizer-induced emission factor of N_2_O for traditional rice cultivation was an average of 1.18%, thus, within the top range of reported emission factors (0.39–1.22%) for rice-cultivation in this climate region^29^. Zou *et al*.^30^ analyzed data on N_2_O emission from Chinese paddy fields (71 measurements from 17 field studies) and observed that depending on water regimes N_2_O emission factors during the rice-growing season averaged 0.42–0.73%. Using a meta-analysis approach for N_2_O emissions from rice fields worldwide, Akiyama *et al*.^31^ & Linquist *et al*.^14^ found that N fertilizer-induced N_2_O emission factor during the rice-growing season ranged from 0.21% to 0.40% across all water management practices, though GCRPS was not included here. The seasonal emission factor of N_2_O in GCRPS under urea broadcast placement (GBP) averaged 3.30%, which is significantly higher than the IPCC default value of 1.0% for global croplands^32^. However, urea deep placement markedly reduced the seasonal emission factor to 1.71% for GDP (Table 1). It should be noted that although GDP substantially decreased N_2_O emissions, its emission factor is still higher than all above published N_2_O emission factors for rice cultivation. In general, as was also observed in our study, N-trace gas emissions were significantly affected by soil inorganic N when the WFPS and soil temperature were not limited in seasonal scale, i.e., soil N_2_O + NO emissions being strongly positively correlated with soil NH_4_^+^ + NO_3_^−^ concentrations^33^. In the GCRPS system, the mean WFPS across the rice season was 75.3–84.2%, which provided the optimal moisture conditions for N_2_O production^28^. At the same time, the high mean soil temperatures under GCRPS (i.e., 24.7–26.0 °C) supported microbial activities. Furthermore, soil nitrogen availability in GCRPS is generally increased as NH_3_ volatilization is hampered due to the coverage of the soil surface with plastic film^34^. Therefore, higher N_2_O emissions from GCRPS could be probably stimulated by more availability of soil NH_4_^+^ and NO_3_^−^, the major factors controlling nitrification and denitrification, respectively. Nevertheless, the trade-offs between NH_3_ volatilization and N_2_O emission under GCRPS practice have not been well investigated to date. In this context, the overall effect of GCRPS on total N_2_O emission could be changed if both indirect N_2_O emission resulting from NH_3_ volatilization^32^ and direct N_2_O emission were taken into account.

To our knowledge, there are still few direct field measurements of NO emissions from paddy fields. Cumulative NO emissions across the rice-growing seasons tended to be higher in 2014 as compared to 2013 across all GCRPS treatments (Table 1). A possible explanation for this inter-annual variation is the difference in seasonal rainfall and its consequent effect on WFPS. The higher rainfall and soil WFPS in 2013 versus 2014 (i.e., rainfall: 638 vs. 537 mm; mean WFPS: 82% vs. 75%) likely resulted in more anaerobic soil conditions, which not only hampers oxidative microbial NO production, but also increases microbial NO consumption in soils^24^. Moreover, the diffusion of NO out of the soil is hampered, resulting in a lowered escape efficiency^24^. All factors together are explaining the lower soil NO emissions in 2013. Across two rice seasons, the fertilizer-induced NO emission factors ranged from 0.008% to 0.112% for all treatments, with mean values of 0.014%, 0.075% and 0.029% for PBP, GBP and GDP, respectively. These values were of a similar magnitude to the estimated NO emission factors of 0.02–0.20% for Chinese rice fields^35–37^, but they were significantly lower than that estimated for Chinese uplands (0.67%)^37^ or the average value of 0.70% for global upland croplands^38^. Overall, based on the results of this and previous studies, it is clear that direct emission factors of N_2_O and NO showed distinct differences among various agricultural management practices. When estimating regional or national inventory of N_2_O and NO emissions, therefore, site- or region-specific emission factors should be used, which also need to consider management practices. Simple extrapolations on the basis of IPCC default values are unlikely to represent regional emissions.

Over two rice seasons, grain yields in all N fertilized treatments ranged from 6.98 to 8.80 Mg dry matter ha^−1^, which is within the range reported for the region^7^. Also, our present results corroborated the previous findings that GCRPS strongly enhanced rice grain yields^8^. Furthermore, our study extended the earlier findings by demonstrating that urea placement methods in GCRPS had significant effect on grain yields, i.e., GDP significantly increased grain yields by 9% on average, compared to GBP (Fig. 3). A possible reason for the higher yields in GDP may be that urea was placed right under and/or beside the rice plants in the GDP plots, which improves overall N use efficiency by increasing opportunities of root access to N. As in this soil depth anaerobic conditions prevail in GCRPS, nitrification is suppressed and the mineral N remains in the form of NH_4_^+^ for plant uptake. Meanwhile, root length and biomass at 0–40 cm soil layer (i.e., root zone) were observed to be greater in GCRPSs than in traditional rice cultivations^9, 34^, which further improves spatial synchrony between crop N demand and soil N availability in GDP.

In our study, the area- and yield-scaled seasonal GHG (CH_4_ plus N_2_O) emissions were estimated to be 3322–4672 (mean: 4186) kg CO_2_-eq ha^−1^ and 522–774 (mean: 646) kg CO_2_-eq Mg^−1^, respectively, for the traditional rice production system (Fig. 5). Our estimates are at the low end of the range of 3500–12080 kg CO_2_-eq ha^−1^ season^−1^ and 649–1290 kg CO_2_-eq Mg^−1^ season^−1^ reported for rice systems in subtropical China^3^. However, if compared to results for drill seeded rice systems in the USA, with GHG emissions being reported to range from 593–6337 kg CO_2_-eq ha^−1^ season^−1^ and 77–784 kg CO_2_-eq Mg^−1^ season^−1^, our estimates are at the top end of the reported range^39^. Similarly, the estimated mean CO_2_-eq emissions for the traditional rice system were comparable to the average value of 3757 kg CO_2_-eq ha^−1^ season^−1^ and 657 kg CO_2_-eq Mg^−1^ for global rice systems^4^. In comparison to the traditional rice production system, GCRPS increased the share of N_2_O emissions on the greenhouse effect when urea was broadcast. But the GHG balance of GCRPS still remained positive as this enhancement of N_2_O emissions were overcompensated by lowered CH_4_ emissions. Consequently, the area- and yield-scaled seasonal GHG emissions for GCRPS were with 654–3346 kg CO_2_-eq ha^−1^ and 112–423 kg CO_2_-eq Mg^−1^, respectively (Fig. 5), generally lower than the above-mentioned values for traditional rice production systems. However, the present CO_2_-eq emissions for GCRPS were comparable to previous estimates for organic rice cropping systems^40^ or rice systems with AWD (alternate wetting and drying) irrigation^41^. Furthermore, the area- and yield-scaled total CH_4_ and N_2_O emissions in GBP were further reduced by GDP which significantly decreased N_2_O emissions and increased grain yields (Tables 1 and 2). Our results show a high potential of GCRPS technology, particularly under urea deep placement for saving irrigation water^9^, increasing rice yield and mitigating GHG (CH_4_ and N_2_O) emission in rice cultivation. Since GDP also significantly reduced the yield-scaled seasonal NO emissions, we postulate that GCRPS with deep placement of urea is an effective climate- and environment-smart technology and management practice to produce more rice for a growing population.

## Methods

### Experimental site and treatments

The experiment was conducted at a rice field (32°07′13″ N, 110°43′04″ E, 440 m above sea level) in the northwest of Hubei province, central China, where the cropping system is dominated by the annual paddy rice-winter fallow rotation. Because of environmental limitations (low spring temperatures and seasonal droughts) for traditional rice cultivations in this region, GCRPS was introduced two decades ago and is now widely adopted by local farmers. The local climate is characterized as northern subtropical monsoon, with a mean annual air temperature of 14.2 °C and an annual precipitation of 914 mm^42^. The soil in the plough layer has a silt loam texture, with 19.8% clay (<0.002 mm), 60.0% silt (0.002–0.05 mm) and 20.2% sand (0.05–2 mm). Other important soil properties are: pH (H_2_O), 6.0; bulk density, 1.36 g cm^−3^; organic C content, 11.9 g C kg^−1^; total N content, 1.31 g N kg^−1^.

The field experiment was performed over the course of two consecutive rice-growing seasons in the years 2013 and 2014, with treatments being arranged in a completely randomized block design with three replicates (each plot with an area of 9.0 m × 10.0 m). A control treatment (GNN, i.e., no N fertilization) and two urea placement methods were established for GCRPS: broadcast placement (GBP) of granular urea (uniformly spread over the soil surface and then incorporated down to 2–3 cm depth) and deep-point placement (DP) of briquetted urea (placed with the appropriate amount of urea into a 10–15 cm deep hole positioned near each rice hill, i.e., with approximately 18 cm × 26 cm spacing). At the N fertilized plots, urea was applied at a rate of 150 kg N ha^−1^ for the rice-growing season. Meanwhile, phosphorus (45 kg P_2_O_5_ ha^−1^ as Ca(H_2_PO_4_)_2_) and potassium (45 kg K_2_O ha^−1^ as KCl) fertilizers were applied for all treatments (i.e., GNN, GBP and GDP) to ensure these nutrients were not limiting. As the film coverage of the soil surface prevents late topdressing, all N, P and K fertilizers were applied as a single dose right before rice transplanting.

As reference, two additional N fertilizer treatments were set up for the traditional paddy rice production system (P): control, 0 kg N ha^−1^ (NN); urea, broadcast placement at 150 kg N ha^−1^ (BP). Except for the water regime which was employed as flooding-midseason drainage-reflooding irrigation until the final drainage before rice harvesting, all other agronomic practices for PNN and PBP were the same as those for GNN and GBP.

The hybrid lowland rice variety Yixiang 3728 was used for all treatments. Before transplanting, each plot in GCRPS was further separated into five raised beds (1.56 m width × 9.4 m length) surrounded by 0.20 m-wide and 0.15 m-deep furrows that were flooded while the raised beds remained uncovered with standing water. All fertilizers were applied as either broadcast or deeply placed into the soil of each plot. For GCRPS the soil surface of all raised beds was covered with a 5 μm transparent polyethylene film, and then two seedlings per hill were transplanted into pre-punched holes at the spacing of 18 cm × 26 cm on April 28, 2013 and April 29, 2014. Rice harvest was done on September 10, 2013 and September 16, 2014. After harvest, rice straw from all treatments was removed from the plots.

### Measurements of GHG and NO fluxes

The fluxes of CH_4_, N_2_O and NO were measured approximately three times per week across the 2013 and 2014 rice-growing seasons, using a static vented chamber-based technique^6, 42^. Flux chambers were composed of a permanent base frame (65 cm width × 90 cm length) that was inserted into 15 cm soil depth, with chamber height varying from 50 to 100 cm throughout the growing season to accommodate plants. For each treatment, the frames were deployed to adequately represent the system. That is, soil management (e.g., soil containing a fertilizer band vs. non-fertilizer band soil) and planting density inside the frames reflected that of the surrounding entire fields, so that gas flux measurements were representative for the respective treatment. The chamber was made of stainless steel and equipped with a vent tube, fan and thermocouple wire^43^.

At each sampling day, gas measurement was carried out between 09:00 am and 11:00 am. The sampling sequence for the different plots was randomized to avoid bias deriving from changing air temperature. Gas samples were taken from the chamber headspace at five equal time (10 min) intervals within 40 min of chamber closure. Concentrations of CH_4_ and N_2_O from the gas samples were analyzed on a gas chromatograph (GC, Agilent 7890 A, Agilent Technoligies, Santa Clara, CA, USA) equipped with a flame ionization detector for CH_4_ detection at 200 °C and an electron capture detector for N_2_O detection at 330 °C^44^. The GC was calibrated using standard CH_4_ and N_2_O provided by the National Center for Standard Matters, Beijing, China. The headspace gas samples were analyzed on a model 42*i* chemiluminescence NO-NO_2_-NO_x_ analyser (Thermo Environmental Instruments Inc., USA) for NO concentrations. The NO_x_ analyser was calibrated monthly by a TE-146*i* dilution-titration instrument with a multi-gas calibrator (dynamic gas calibrator). The fluxes of CH_4_, N_2_O and NO were calculated from the slope of the linear or nonlinear model fitted to the concentration of each gas against the chamber closure time^42, 45^. Seasonal cumulative emissions of CH_4_, N_2_O and NO were directly computed from the measured fluxes and were estimated by linear interpolation between measuring days. It should be noted here that manual gas flux measurements with intensive sampling schedule allows to reliably reflecting the temporal variability of soil trace gas fluxes, while it may either over- or under-estimate the cumulative emissions over a given period. For example, an earlier comparison of manual with automated soil GHG emissions for a cultivated land showed that cumulative emissions of N_2_O in terms of manual chamber measurements might be overestimated by approximately 18%^46^.

In addition, soil CH_4_, N_2_O and NO fluxes were also measured from each treatment during the fallow period from September 2013 to April 2014 (approximately 227 days). The detection and calculation methods were the same as described above.

### Auxiliary measurements

Air temperature and rainfall data during the experimental period were obtained from an on-site weather station (WeatherHawk 500, Campbell Scientific, USA). Soil (5 cm) temperature was automatically recorded in 30 min intervals using a HOBO temperature sensor (Onset, USA). Soil volumetric water content at 0–6 cm depth in each GCRPS plot was measured daily using a frequency domain reflectometer (MPM-160, China). Three samples from the topsoil (0–10 cm) of each plot were taken at 1–2 week intervals across the experimental period to determine exchangeable ammonium (NH_4_^+^) and nitrate (NO_3_^−^). Following the sampling, fresh soil samples were extracted with 1 M KCl solution in a soil:solution ratio of 1:5, and the extracts were analyzed on a continuous flow analyzer (San++, Skalar Analytical B.V., Netherlands) for NH_4_^+^ and NO_3_^−^ concentrations. At physiological maturity, rice plants from a 10 m^2^ area within the center of each plot was manually harvested close to the soil surface, separated into straw and grain components, and dried at 70 °C to a constant weight.

### Statistical analysis

Aggregate emission of CH_4_ and N_2_O was calculated in mass of CO_2_ equivalents (CH_4_: 34; N_2_O: 298) over a 100-yr time horizon^1^. Yield-scaled emission of CH_4_, N_2_O, NO or CH_4_ + N_2_O was computed by taking the ratio of seasonal emission and grain yield.

All statistical analyses were performed using the SPSS software for Windows (SPSS 19.0, SPSS China, Beijing, China). Prior to variance component analysis, all data or log-transformed data were subjected to normality tests. Significant differences between rice production system, N fertilizer treatment, year and their interactions for trace gas fluxes were determined using Linear Mixed Models on the basis of a randomized complete block design. Differences in seasonal gas emissions and crop yields among the combined rice cultivation and N fertilizer treatments were further examined by the Turkey’s multiple range test. The correlation between trace gas fluxes and soil environmental variables (i.e., soil water content, temperature and inorganic N content) was examined by multiple linear regression analyses with the stepwise procedure. Significant differences/relationships were given at P < 0.05 level.

## Electronic supplementary material

Supplementary Information
