# Supplementary material for: Urea deep placement reduces yield-scaled greenhouse gas (CH4 and N2O) and NO emissions from a ground cover rice production system
Source: Sci Rep. 2017 Sep 12;7:11415. doi: 10.1038/s41598-017-11772-2 (PMC5595888; doi:10.1038/s41598-017-11772-2)
Supplement: Supplementary file 1 — Supplementary Information [file 41598_2017_11772_MOESM1_ESM.pdf]

Supplementary Information

Urea deep placement reduces yield-scaled greenhouse gas (CH_4 and N_2O) and NO emissions from a ground cover rice production system

Zhisheng Yao[1,2,*], Xunhua Zheng[1,3], Yanan Zhang[4], Chunyan Liu[1], Rui Wang[1], Shan Lin[4], Qiang Zuo[4], Klaus Butterbach-Bahl[2]

[1] State Key Laboratory of Atmospheric Boundary Layer Physics and Atmospheric Chemistry, Institute of Atmospheric Physics, Chinese Academy of Sciences, Beijing 100029, P.R. China

[2] Institute for Meteorology and Climate Research, Atmospheric Environmental Research, Karlsruhe Institute of Technology, D-82467 Garmisch-Partenkirchen, Germany

[3] College of Earth Science, University of Chinese Academy of Sciences, Beijing 100049, P.R.China

[4] College of Resource and Environmental Science, China Agricultural University, Beijing 100193, P.R. China

* Corresponding author

Name: Zhisheng Yao

Tel: 0086-10-62025885

Fax: 0086-10-62041393

E-mail: zhishengyao@mail.iap.ac.cn

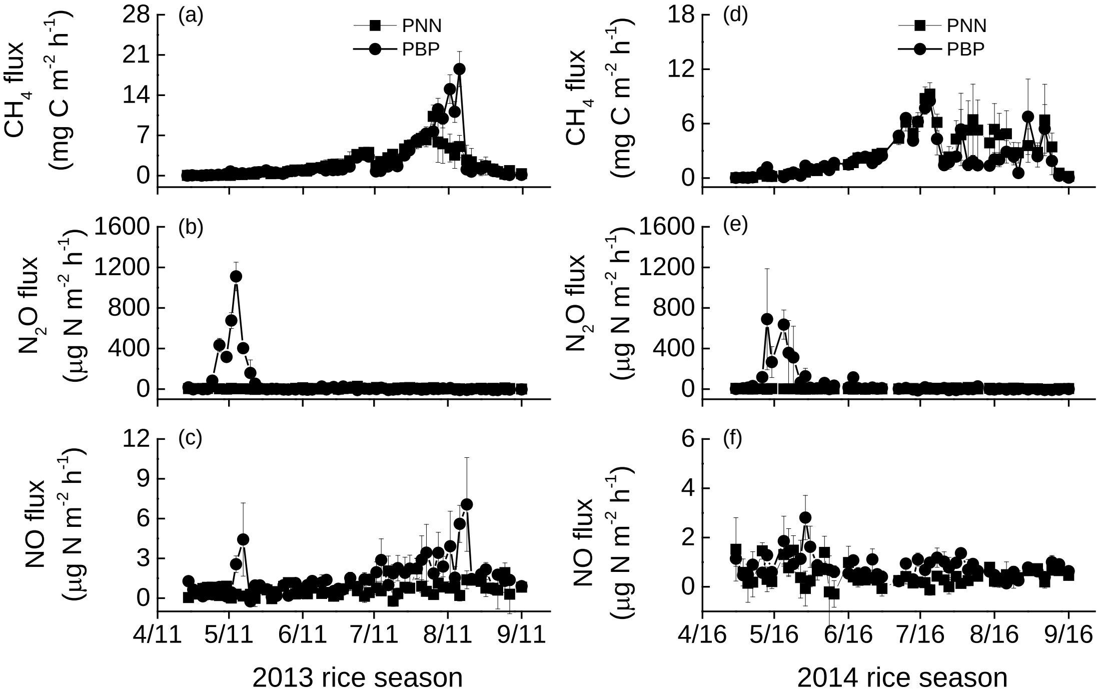

Figure S1. Seasonal variations of (a, d) methane (CH_4), (b, e) nitrous oxide (N_2O), and (c, f) nitric oxide (NO) fluxes from the traditional paddy rice production system under different fertilizer treatments (i.e., no N fertilization (PNN) and broadcast placement of urea (PBP)) during the rice-growing seasons of 2013 and 2014.

Table S1 ANOVA analysis for the effects of rice production system (RS), N fertilizer treatment (F) and Year (Y) on the seasonal fluxes of methane (CH_4), nitrous oxide (N_2O) and nitric oxide (NO) as well as total CH_4+N_2O emissions during the experimental period

Factors	df	CH_4 (kg C ha^{-1})			N_2O (kg N ha^{-1})			NO (kg N ha^{-1})			CH_4+N_2O (kg CO_2-eq ha^{-1})		
		SS	*F*	*P*	*SS*	*F*	*P*	*SS*	*F*	*P*	*SS*	*F*	*P*
RS	1	24845	56.2	<0.001	15.1	36.3	<0.001	0.020	5.97	<0.05	2.83E7	30.1	<0.001
F	2	291	0.329	0.72	67.5	80.9	<0.001	0.028	4.27	<0.05	9.95E6	5.29	<0.05
Y	1	39.5	0.089	0.77	5.43	13.0	<0.05	0.003	1.02	0.33	649097	0.690	0.42
RS×F	1	39.5	0.089	0.77	15.2	36.4	<0.001	0.013	3.84	0.064	2.37E6	2.52	0.13
RS×Y	1	819	1.85	0.19	2.91	6.98	<0.05	0.009	2.60	0.12	247457	0.263	0.61
F×Y	2	227	0.257	0.78	3.50	4.19	<0.05	0.003	0.49	0.62	325366	0.173	0.84
RS×F×Y	1	504	1.14	0.30	2.82	6.75	<0.05	0.006	1.96	0.18	3259751	3.46	0.078
Model	9	33019	8.29	<0.001	119	31.7	<0.001	0.085	2.88	<0.05	5.17E7	6.11	<0.001
Error	20	8845			8.34			0.066			1.88E7		

Table S2 Seasonal cumulative emissions of methane (CH_4, in kg C ha^{-1}), nitrous oxide (N_2O, in kg N ha^{-1}) and nitric oxide (NO, kg N ha^{-1}) for various agronomic treatments during the fallow season of 2013-2014 (i.e., approximately 227 days from September 2013 to April 2014)

Season	Code[†]	CH_4[*]	N_2O[*]	NO[*]
2013-2014 fallow season				
	PNN	-1.02±0.17 a	0.90±0.37 a	0.14±0.01 a
	PBP	-0.55±0.08 a	2.29±1.36 a	0.17±0.02 a
	GNN	-0.59±0.03 a	0.48±0.07 a	0.11±0.02 a
	GBP	-0.63±0.07 a	0.71±0.07 a	0.15±0.02 a
	GDP	-0.75±0.08 a	0.63±0.02 a	0.15±0.01 a

[†] PNN, no N fertilization in the traditional paddy rice production system; PBP, broadcast placement of urea at a common rate of 150 kg N ha^{-1} in the traditional paddy rice production system; GNN, no N fertilization in the ground cover rice production system; GBP, broadcast placement of urea at a common rate of 150 kg N ha^{-1} in the ground cover rice production system; GDP, deep-point placement of urea at a common rate of 150 kg N ha^{-1} in the ground cover rice production system. * The same letters within the same column indicate no significant differences among N fertilizer treatments at the $P < 0.05$ level.
